# Gait Initiation Impairment in Patients with Parkinson’s Disease and Freezing of Gait

**DOI:** 10.3390/bioengineering9110639

**Published:** 2022-11-02

**Authors:** Chiara Palmisano, Laura Beccaria, Stefan Haufe, Jens Volkmann, Gianni Pezzoli, Ioannis U. Isaias

**Affiliations:** 1 Department of Neurology, University Hospital and Julius-Maximilian-University, 97080 Würzburg, Germany; 2 Uncertainty, Inverse Modeling and Machine Learning Group, Faculty IV Electrical Engineering and Computer Science, Technical University of Berlin, 10623 Berlin, Germany; 3 Centro Parkinson, ASST Gaetano Pini-CTO, 20122 Milano, Italy

**Keywords:** freezing of gait, gait initiation, Parkinson’s disease, posture, segmental centers of mass, anthropometric measurement, base of support

## Abstract

Freezing of gait (FOG) is a sudden episodic inability to produce effective stepping despite the intention to walk. It typically occurs during gait initiation (GI) or modulation and may lead to falls. We studied the anticipatory postural adjustments (imbalance, unloading, and stepping phase) at GI in 23 patients with Parkinson’s disease (PD) and FOG (PDF), 20 patients with PD and no previous history of FOG (PDNF), and 23 healthy controls (HCs). Patients performed the task when off dopaminergic medications. The center of pressure (CoP) displacement and velocity during imbalance showed significant impairment in both PDNF and PDF, more prominent in the latter patients. Several measurements were specifically impaired in PDF patients, especially the CoP displacement along the anteroposterior axis during unloading. The pattern of segmental center of mass (SCoM) movements did not show differences between groups. The standing postural profile preceding GI did not correlate with outcome measurements. We have shown impaired motor programming at GI in Parkinsonian patients. The more prominent deterioration of unloading in PDF patients might suggest impaired processing and integration of somatosensory information subserving GI. The unaltered temporal movement sequencing of SCoM might indicate some compensatory cerebellar mechanisms triggering time-locked models of body mechanics in PD.

## 1. Introduction

Freezing of gait (FOG) is a dramatic phenomenon frequently affecting patients with Parkinson’s disease (PD) [1], causing falls, mobility restrictions, and poor quality of life [2,3,4]. FOG is defined as a brief, episodic absence or marked reduction of forward progression of the feet despite the intention to walk [5], which typically occurs when initiating or modulating gait (e.g., turning, obstacle crossing, and so on).

Gait initiation (GI) is a highly challenging task for the balance control system and is of particular interest in the study of neural control of upright posture maintenance during whole-body movement [6]. Specifically, this task allows the precise assessment of anticipatory postural adjustments (APAs; i.e., muscular synergies that precede GI), aiming to destabilize the antigravity postural set by shifting the center of pressure (CoP) to generate a gravitational moment favoring the center of mass (CoM) forward acceleration [7]. APAs are considered a motor program controlled by feedforward mechanisms regulated by the supraspinal locomotor network [8,9,10,11,12]. The selection and scaling of appropriate APAs rely on the ability to use sensory information to determine the body positioning relative to the environment prior to step execution [13,14] and on the intended forthcoming movement (natural, slow, fast, obstacle, and so on) [7,15,16,17]. Striatal dopamine loss, a pathophysiological hallmark of PD, greatly impacts the production of APAs at GI and particularly the CoP displacement and velocity [12]. Only a few studies have specifically investigated the GI task in Parkinsonian patients with a history of FOG (PDF), non-implanted for deep brain stimulation (DBS), and after withdrawal of dopaminergic medication (meds-off state). The stimulation and medication condition should be carefully considered, as both DBS and dopaminergic drugs can variably influence posture and gait in PD [12,18,19,20,21,22,23,24,25,26]. Overall, these studies showed conflicting results, with APAs being reported as normal [27,28,29] or multiple and hypometric [10,30]. Several methodological discrepancies may account for such different findings, including a non-standardized meds-off state [27], imposed (predefined) feet positioning [29], cueing [10,29,30], and specific instructions on the execution of the GI task (e.g., to start walking as quickly as possible [30,31] or while performing a cognitive task [28]). All of these factors can significantly impact and alter APA expression at GI. Specifically, a cued start signal influences motor programming towards normalization, especially in PDF [9,18], similar to the improvements seen with the administration of levodopa for self-generated step initiation [18]. Moreover, the initial feet position [12,22,32] and posture [33,34,35] can significantly impact the biomechanical features of APAs at GI.

Postural changes in particular would have a detrimental impact on APA production. An altered representation of the body position (egocentric representation) may determine a functional re-organization of the supplementary motor area (SMA)-proper, hampering selection and re-scaling of APAs to adapt to the altered postural framework and bradykinetic stepping [33,34,36,37,38].

Our study aims to describe GI alteration in patients with PD and FOG, accounting for the influence of anthropometric measurements (AMs) and the base of support (BoS) and investigating their relationship with the initial posture. We have also addressed the relative timing and movement sequence of each body segment subserving GI.

## 2. Materials and Methods

### 2.1. Subjects

We recruited 23 patients with idiopathic PD (according to the U.K. Brain Bank criteria) and an unambiguous, previous history of FOG (PDF; i.e., patients reporting episodes of FOG on a daily basis prior to the experiment). On the day of the experiment, the presence of FOG was confirmed with a clinical evaluation by an experienced neurologist (I.U.I.). In addition, 20 patients with PD and no previous history of FOG (PDNF) and 23 healthy controls (HCs) were also included. HCs and PDNF patients were chosen to match in terms of demographic and clinical data with the PDF group. Subjects with neurological diseases other than PD, including cognitive decline (i.e., Mini-Mental State Examination score < 27), vestibular disorders, and orthopedic impairments that could interfere with gait were excluded. Disease severity was evaluated with the Unified Parkinson’s Disease Rating Scale motor part (UPDRS-III).

### 2.2. Experimental Protocol

Patients were investigated in practical meds-off state, i.e., in the morning after overnight withdrawal (>12 h) of all dopaminergic drugs.

Kinematic data were recorded using an optoelectronic system with six cameras (sampling rate 60 Hz, SMART 1.10, BTS, Garbagnate Milanese, Italy) and a set of 29 markers placed on anatomical landmarks (temples, acromions, lateral humeral condyles, ulnar styloids, anterior superior iliac spines, middle thighs, lateral femoral condyles, fibula heads, tibial anterior side, lateral malleoli, Achilles tendon insertion, fifth metatarsal heads, halluxes, the seventh cervical vertebra [C7], point of maximum kyphosis, and middle point between the posterior superior iliac spines) [39,40]. Eight additional technical markers were placed on the trochanters, the medial condyles, the medial malleoli, and the first metatarsi for a short calibration trial, which allowed the computation of the AMs and BoS measurements [11,12,41]. Markers traces were filtered with a fifth-order lowpass Butterworth filter (cut-off frequency: 10 Hz [41]). Dynamic measurements were recorded with a force plate working at a sampling rate of 960 Hz (KISTLER 9286A, Winterthur, Switzerland). The resulting signal was low-pass filtered (fifth-order lowpass Butterworth filter) with a cut-off frequency of 30 Hz [11,42].

At the beginning of each trial, subjects stood upright on the force platform at a comfortable stance position for about 30 s. The initial stance position was not standardized to prevent modification of the subject’s usual motor strategy to initiate gait [12].

Participants were instructed to start walking after a self-selected period from a verbal signal, in order to avoid any effect of cueing on GI. The instruction given was as follows: “Start walking at the moment of your choice”. Subjects were not instructed on the stepping leg to use and they moved at their own pace until the end of the walkway. After a training session, at least three consecutive trials were recorded. The principal investigator supervised all participants during the experiment.

### 2.3. Biomechanical Measurements

#### 2.3.1. Anthropometric Measurements and Base of Support

For each subject, we measured the following AMs (Table 1): body height, inter anterior-superior iliac spine distance, limb length, foot length, body mass, and body mass index. The AMs were recorded over a period of 5 s of standing using eight additional markers, as described in [12]. The AMs were used for the estimation of the CoM of each body segment (SCoM), according to the anthropometric tables and regression equations proposed by [43]. For each trial, the BoS area and BoS width were calculated. We also accounted for feet position asymmetry by measuring the foot alignment, the difference between feet extra-rotation angles, and the BoS opening angle [11,12].

#### 2.3.2. Postural Profile

**Figure 1:**
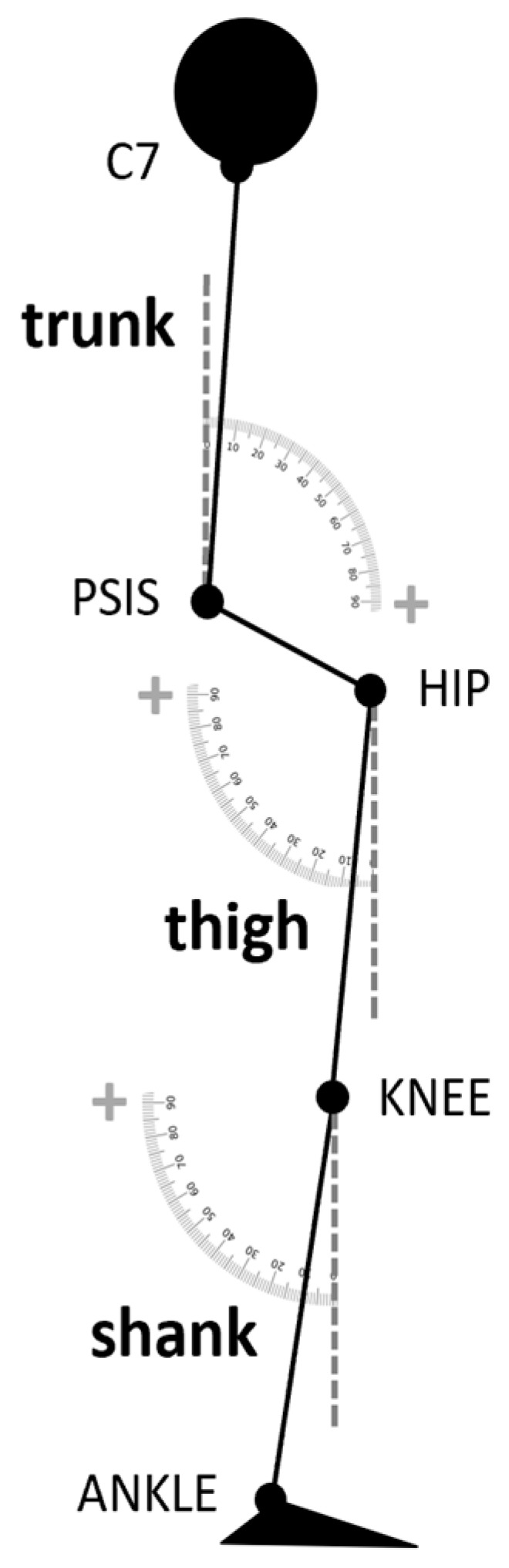
Scheme of the postural angles analyzed in the study. The trunk angle was defined as the inclination of the line passing through the markers placed on the middle point between the two posterior superior iliac spines and the seventh cervical vertebra with respect to the vertical axis of the laboratory. The thigh angle was calculated as the angle between the vector connecting the knee and hip center of rotation and the vertical axis of the laboratory. The shank angle was computed between the line connecting the joint centers of the knee and ankle and the vertical axis of the laboratory.

The standing postural profile was characterized by means of trunk, thigh, and shank sagittal angles (Figure 1) [20] computed shortly before the GI execution (during a 1 s window before the onset of the APAs). The trunk angle was defined as the inclination of the line passing through the markers placed on the middle point between the two posterior superior iliac spines and the seventh cervical vertebra with respect to the vertical axis of the laboratory. The thigh angle was calculated as the angle between the vector connecting the knee and hip center of rotation and the vertical axis of the laboratory. The shank angle was computed between the line connecting the joint centers of the knee and ankle and the vertical axis of the laboratory.

#### 2.3.3. Anticipatory Postural Adjustments and Gait Initiation

**Figure 2:**
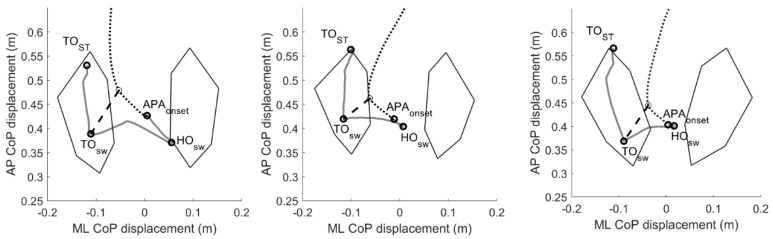
Two-dimensional center of pressure and center of mass trajectories during gait initiation. Example of the pathway of the center of pressure (CoP, grey solid line) and center of mass (CoM, black dotted line) during a gait initiation trial of one healthy subject (left panel) and one Parkinsonian patient without (PDNF, central panel) and one patient with (PDF, right panel) a positive history of freezing of gait. We defined the imbalance (IMB) phase as the interval between the onset of the APAs (APAONSET) and the heel-off of the swing foot (HOSW), and the unloading phase (UNL) as the interval between the HOSW and the toe-off of the swing foot (TOSW). The black dashed line represents the CoP–CoM vector at the end of the unloading (UNL) phase. With respect to healthy controls, the CoP displacement during the IMB phase was reduced for both PD and PDF patients. The CoP displacement during the UNL phase was in most cases backwards for the PDF patients only. Please see Table 3 for further details. Abbreviations: APAs, anticipatory postural adjustments; AP, anterior–posterior; CoP, center of pressure; HC, healthy controls; HO, heel off; ML, mediolateral; PDF, Parkinson’s disease with freezing of gait; PDNF, Parkinson’s disease with no freezing of gait; TO, toe-off.

GI variables were defined based on the displacement of the CoP, recorded by the force platform. The CoM was estimated as the weighted mean of the SCoM [44]. GI variables were calculated by dedicated algorithms in Matlab ambient (Matlab^®^ R2018b, The MathWorks Inc., Natick, MA, USA) (as in [11,12]). All GI measurements computed in the study are listed and described in Table 1. Briefly, four reference instants were automatically identified on the CoP track and checked by visual inspection using an interactive software: the onset of the APAs, the heel-off of the swing foot (HO_SW_), the toe-off of the swing foot (TO_SW_), and the toe-off of the stance foot (TO_ST_). The APA onset (APA_ONSET_) was detected as the instant at which the CoP started moving consistently backward and toward the swing foot; HO_SW_ was defined as the time at which CoP reached the most lateral position toward the swing foot; TO_SW_ was defined as the moment at which the CoP shifted from lateral to anterior motion; and TO_ST_ was defined as the last frame of the force platform signal (Figure 2). The APAs were divided into two periods: the imbalance phase (IMB), from APA_ONSET_ to HO_SW_, and the unloading phase (UNL), from HO_SW_ to TO_SW_ [12,20,40,45]. The following measurements were calculated for both the IMB and UNL periods: duration and anteroposterior and mediolateral CoP displacement, average velocity, and maximal velocity (Table 1). Of note, the mediolateral CoP displacement during the imbalance phase was considered positive when the shift of the CoP was towards the swing foot, while the mediolateral CoP displacement during the unloading phase was considered positive when the CoP was moving towards the stance foot. The IMB and UNL anteroposterior CoP displacement were both defined as positive when the CoP movement was oriented backwards. We additionally defined the stepping phase, from HO_SW_ to the subsequent heel contact of the swing foot, by means of markers placed on the feet. The first step was characterized in terms of step length and average and maximal velocity (Table 1). Velocity and acceleration of the CoM were defined at the end of the IMB and UNL phases and at the instant of TO_ST_. Additionally, the position of the CoM with respect to the CoP and the inclination of the vector connecting the two points in the transversal plane were computed at the end of IMB and UNL and at the TO_ST_ (Table 1) [12].

#### 2.3.4. Segmental Centers of Mass

To describe the temporal pattern of segmental movements during GI, we computed the latency of movement onset of the following 16 SCoM: head, chest, abdomen, pelvis, swing arm, stance arm, swing forearm, stance forearm, swing hand, stance hand, swing thigh, stance thigh, swing shank, stance shank, swing foot, and stance foot (similarly to [46]). For each trial, the movement onset latency of each SCoM was computed as the movement time from the onset of the APAs and normalized for the total GI time (from APA_ONSET_ to the toe-off of the swing foot). For each subject, we rank-ordered the SCoM onset times and computed the following for each group: (i) the movement time from APA_ONSET_ normalized for the total GI time and (ii) the relative frequency of each SCoM onset time to appear as events 1–16 of GI. To improve the readability of the data, we repeated the analysis after combining the SCoM into six groups (upper trunk: head and chest; lower trunk: abdomen and pelvis; swing arm: swing arm, forearm, and hand; stance arm: stance arm, forearm, and hand; swing leg: swing thigh, shank, and foot; and stance leg: stance thigh, shank, and foot).

### 2.4. Statistical Analysis

For each subject, all measurements were averaged over GI trials executed with the same swing foot. Each participant performed at least three GI trials with the same swing foot. Single trials and average values were inspected and outliers were removed from further analyses based on the Mahalanobis distance [47,48].

First, we verified matching between groups for demographic, clinic, BoS, and AM features with a Mann–Whitney U-test (*p*-value set at 0.05). Before comparing the GI measurements across groups, we investigated their relationship with the BoS and AMs with two partial correlation analyses [12]. For each group, we correlated the GI measurements first with the BoS measurements controlling for the AMs, and then with the AMs controlling for the BoS. In agreement with [11], GI variables that significantly correlated (Spearman’s ρ > 0.5 and *p*-value < 0.01) with the BoS in at least one group were excluded from further analyses. We opted for this conservative approach because the BoS was freely chosen by the subjects and may have been influenced by both the disease and compensatory mechanisms. The GI variables that correlated (Spearman’s ρ > 0.5 and *p*-value < 0.01) with the AMs were instead corrected by means of the decorrelation normalization technique, as described by O’Malley [49]. This correction was applicable as AMs were not influenced by the disease (no patient had camptocormia, skeletal deformities, and so on).

GI variables not dependent on the BoS and decorrelated from the influence of the AMs were then compared between groups using a Dunn’s test (*p*-value set at 0.05, adjusted with Bonferroni correction for multiple comparisons).

We then investigated alterations of the initial postural condition. As for the GI measurements, we assessed the correlation of the AMs and the BoS with the postural angles with partial correlation analyses (Spearman’s ρ > 0.5 and *p*-value < 0.01), before comparing the postural angles across groups (Dunn’s test, *p*-value set at 0.05, adjusted with Bonferroni correction for multiple comparisons).

As we found differences in the postural profiles across groups, we investigated whether altered GI measurements in the PD groups were related to postural changes rather than to impaired motor programming. We performed a partial correlation analysis between the GI outcome measurements and the postural angles correcting for the group variable. We considered a correlation significant when Spearman’s ρ > 0.5 and *p*-value < 0.01.

Differences across groups in the SCoM movement onset were analyzed with a Dunn’s test (*p*-value < 0.05, adjusted with Bonferroni correction for multiple comparisons).

All statistical analyses, except partial correlation analyses performed in Matlab, were performed with the JMP package (JMP^®^ Pro 14.0.0, SAS Institute Inc., Cary, NC, USA).

## 3. Results

Demographic features, AM measurements, and BoS measurements did not significantly differ between groups (Table 2). Clinical data were similar between PDNF and PDF patients (Table 2).

Of note, none of the patients showed freezing episodes during GI recordings. Therefore, our results define primarily the impact of APA alterations and postural features in favoring FOG in PD and not a causal correlation with the occurrence of gait freezing episodes at GI.

### 3.1. Selection of GI Variables

The BoS did not correlate with most of the biomechanical measures of the IMB and stepping phases, but did correlate with the UNL. The results are consistent with our previous findings [12]. The GI variables that were independent from the BoS are listed in Table 3. The BoS and the AMs showed no correlations with the trunk, thigh, and shank angles.

**Table 3:** Gait initiation measurements: comparison between groups. Only biomechanical variables not correlated with the base of support are listed. Data are shown as mean (standard deviation). The mediolateral CoP displacement during imbalance and unloading was considered positive when the shift in the CoP was towards the swing and the stance foot, respectively. The anteroposterior CoP displacement during imbalance and unloading phase were both defined as positive when the CoP movement was oriented backwards. Abbreviations: HC, healthy controls; PDF, Parkinson’s disease with freezing of gait; PDNF, Parkinson’s disease with no freezing of gait; refer to Table 1 for a list of other acronyms used.

	HC	PDNF	PDF
IMB duration (s)	0.39 (0.08)	0.38 (0.08)	0.33 (0.09)
IMB displacement (mm)	61.23 ^+^ (20.32)	35.54 (19.71)	23.67 ^+^ (9.94)
IMB displacement ML (mm)	42.07 ^#,+^ (13.04)	24.04 ^#^ (13.61)	17.76 ^+^ (8.59)
IMB displacement AP (mm)	36.53 ^+^ (16.17)	18.79 (14.75)	9.16 ^+^ (5.93)
IMB average velocity (mm/s)	163.40 ^#,+^ (62.29)	90.79 ^#^ (47.29)	84.03 ^+^ (46.81)
IMB average velocity ML (mm/s)	110.94 ^#^ (34.90)	62.14 ^#^ (34.80)	67.53 (41.47)
IMB average velocity AP (mm/s)	103.36 ^#,+^ (53.78)	47.74 ^#^ (34.39)	40.19 ^+^ (30.45)
IMB maximal velocity (mm/s)	344.22 ^#,+^ (149.41)	189.88 ^#^ (113.54)	150.41 ^+^ (64.26)
IMB maximal velocity ML (mm/s)	238.29 ^#,+^ (77.82)	137.25 ^#^ (72.76)	124.97 ^+^ (56.96)
IMB maximal velocity AP (mm/s)	225.81 ^+^ (110.67)	124.78 (65.52)	101.41 ^+^ (55.74)
IMB end CoM velocity (m/s)	0.09 ^+^ (0.03)	0.06 (0.03)	0.04 ^+^ (0.02)
IMB end CoP–CoM distance (m)	0.07 ^+^ (0.02)	0.04 (0.02)	0.03 ^+^ (0.01)
UNL duration (s)	0.36 (0.08)	0.40 (0.08)	0.45 (0.19)
UNL displacement AP (mm)	−9.67 ^+^ (15.30)	−6.25 * (18.22)	14.69 ^+,^* (14.70)
UNL average velocity (mm/s)	465.61 (162.21)	323.94 (131.02)	320.34 (150.20)
UNL average velocity ML (mm/s)	422.79 (148.96)	289.26 (121.24)	290.67 (140.81)
UNL average velocity AP (mm/s)	53.11 (20.97)	37.29 (17.16)	46.04 (34.21)
UNL maximal velocity AP (mm/s)	344.48 (154.35)	388.76 (169.88)	359.19 (178.76)
UNL end CoM velocity (m/s)	0.21 ^+^ (0.06)	0.16 (0.07)	0.11 ^+^ (0.04)
UNL end CoM acceleration (m/s^2^)	1.29 (0.33)	1.08 (0.41)	1.12 (0.25)
UNL end CoP–CoM distance (m)	0.08 (0.03)	0.07 (0.03)	0.07 (0.02)
ST toe-off CoM velocity (m/s)	0.86 ^#,+^ (0.13)	0.63 ^#^ (0.24)	0.53 ^+^ (0.18)
ST toe-off CoM acceleration (m/s^2^)	1.73 ^+^ (0.38)	1.28 (0.42)	1.08 ^+^ (0.33)
ST toe CoP–CoM distance (m)	0.48 (0.32)	0.51 (0.29)	0.34 (0.28)
First step length (m)	0.56 ^+^ (0.07)	0.43 (0.14)	0.33 ^+^ (0.13)

Dunn’s test, significant *p*-value after Bonferroni correction: ^#^ HC vs. PDNF, ^+^ HC vs. PDF, * PDNF vs. PDF.

### 3.2. Postural Features

The trunk and thigh angles, but not the shank angle, were significantly altered in both PDNF and PDF patients compared with HCs (Table 4). Parkinsonian patients showed increased forward trunk bending associated with a reduced thigh angle. The trunk was more flexed in PDF patients than in PDNF patients, although this difference did not reach statistical significance. The thigh angle showed a negative average value only in the PDF group.

### 3.3. Effect of PD and History of FOG on GI

We observed significant alterations in the GI execution in both PDNF and PDF patients, with the latter group showing overall more severely altered APA measurements (Table 3, Figure 2).

The CoP displacement and velocity during IMB showed a progressive and significant reduction from HCs to PDNF to PDF groups along both the mediolateral and anteroposterior axes.

The UNL and stepping phases were also altered in PDNF and PDF patients (Table 3, Figure 2). Of most relevance, in PDF patients, the anteroposterior displacement of the CoP during UNL was backwards in most of the trials.

PDF patients had a significantly reduced first step length and both PD groups had a lower first step average velocity compared with HCs.

The CoM forward propulsion (velocity and acceleration) progressively decreased from HCs to PDNF to PDF.

### 3.4. Relationship between the Standing Postural Profile and the GI

We did not find any significant correlation between the postural angles and the GI measurements. However, when not correcting for multiple comparisons, the shank angle was predictive for velocity variables of the IMB phase. The results are shown in Table 5.

### 3.5. Pattern of Movements during GI

The overall pattern of segmental movements during GI did not show clear differences between groups (Table 6 and Table 7). However, PDF showed shorter times of movement onset for almost all ranked segments (Table 7), possibly suggesting tight inter-segmental coupling [50]. All groups started preferably with the swing or stance arm, especially the swing hand for HCs and PDNF and the stance hand for PDF (Figures S1 and S2). The abdomen was often the last body segment moved by HCs and PDNF, but not by PDF (Figure S1). Of note, we observed a remarkable inter-subject variability of SCoM onset times, especially for PD, as shown by the high value of the standard deviation (Table 6) and the large dispersion of the temporal order of SCoM movement onsets (Figure S1), which probably prevented us from capturing statistically significant differences.

## 4. Discussion

This study aimed to evaluate the specific biomechanical alterations of APAs at GI in PD patients with a positive history of FOG, accounting for known confounders such as medication condition, anthropometric measurements, base of support, and initial stance posture. The CoP displacement and velocity during the imbalance phase were altered in both PDNF and PDF patients, but more prominently in the latter group. The CoP displacement along the anteroposterior axis during the unloading phase was impaired only in PDF patients. The order of SCoM movements was unaltered in the two patient groups. The postural profile did not correlate with GI outcome measurements.

Our findings are in line with previous studies in PD that showed an impairment in APAs’ production at GI [20,51]. However, a direct comparison with earlier works is limited because we aimed to minimize possible bias from cueing or imposed postural constraints that are known to affect the execution of the GI task [9,11,12,18,22,52,53,54].

We have now shown that there is a profound alteration of APA execution in PDF patients, which cannot be attributed to specific demographic or clinical features (such as disease severity and duration, medication dose, and efficacy) as the patient groups were matched for all of these features [55].

The IMB phase of APA execution was significantly altered in all PD patients, particularly in PDF (Table 3). Increasing evidence suggests that this GI phase is governed by centrally mediated feedforward signals and involves the cortico-basal ganglia loop, with the SMA-proper and the striatum chiefly contributing to the execution of these pre-programmed movements [6,8,9,11,12,56,57,58,59,60]. In PD, we have previously shown a detrimental effect of striatal dopamine loss in the IMB execution at GI [12]. Recent studies in Parkinsonian patients suggested that striatal dopamine may in part enable normal movement by encoding sensitivity to the energy cost of a movement [61,62,63,64]. Therefore, from the perspective of motor planning, especially of patterned and consolidated motor actions such as APAs, a reduced tonic dopaminergic activity could reframe the coding of the expected energetic costs and impair motor control [63].

In our study, we also showed a prominent alteration in the AP displacement of the CoP during the UNL phase in PDF patients. We interpret this result as a possible alteration, mainly of PDF patients, in the processing and integration of somatosensory information prior to stepping [6,14,65,66]. A chief contribution to integrate proprioceptive and voluntary components for a proper weight transfer during GI can be expected from the premotor–parietal–cerebellar loop [14,58,67,68,69,70,71]. An impaired ability to inhibit stance postural control and initiate stepping and poor set-shifting is also included in pathophysiological hypotheses of FOG in PD [5,10,57,72,73,74,75,76,77].

Despite impaired APA execution, the sequencing of the movement did not show major alterations in the PD groups. We speculate that additional inputs from the cerebellum could overcome impaired information processing by favoring internal movement timing [78]. The efficacy of an online compensatory role of the cerebellum [70,78] is suggested in our study by the relatively preserved SCoM temporal movement sequencing [79], which could have also prevented the appearance of any gait freezing episode during our acquisitions. Relative timing of segmental movements was also described as unaltered in patients with PD by Rosin and colleagues (1997), further suggesting a compensatory rather than detrimental role of the cerebellum in Parkinsonian patients with FOG and balance disturbances [60,78,80]. Of relevance, the high variability in the SCoM movement onsets might have prevented us from detecting differences across groups. Further studies with larger cohorts might further explore this aspect to definitively rule out the presence of PD-related alterations in the movement sequencing.

We envisioned a significant impact of postural abnormalities on GI in PD, but our results did not support this hypothesis. Interestingly, our findings instead confirmed previous physiological studies reporting no correlation between APA execution at GI and the natural inclination of the trunk [33] or of a forward leaning up to 30% of the maximum voluntary lean [35].

Our study suffers from some limitations. First, although we reduced as much as possible the influence of known confounders (i.e., initial feet position and posture, anthropometric parameters, and cues), we cannot fully exclude a residual influence of Parkinsonian symptoms such as bradykinesia and rigidity on the task performance [37]. However, in our previous work [12], we showed that levodopa intake, by improving bradykinesia and rigidity, increases the length and speed of the first step at GI, but does not affect the AP shift during UNL. We can thus hypothesize that the alterations in AP displacement during UNL in the PDF group are not related to akinetic-rigid symptoms, but to impairment of the motor program itself. Future studies are needed to better clarify this aspect. Second, the limited sample size and very stringent statistics may have limited the detection of differences between groups (e.g., SCoM onset times). Third, the lack of a brain imaging evaluation in this study prevents any firm conclusions about our pathophysiological interpretation of the kinematic and dynamic findings, but they match well with the brain metabolic activity changes [66,68] and network derangements [81,82,83,84] described during actual gait and gait freezing episodes in Parkinsonian patients.

In conclusion, our data demonstrate substantial impairment of feedforward motor programming mechanisms at GI in Parkinsonian patients. The deterioration of the UNL and stepping in PDF patients would suggest an additional impaired integration of postural and locomotor programs subserving gait initiation and modulation, which might be partly compensated by cerebellar mechanisms triggering time-locked models of body movement. Postural alterations seem to play a minor role in GI impairment in patients with PD. Last, but not least, our results suggest the potential clinical utility of recording the CoP displacement during GI, and particularly its AP shift during the UNL to identify patients at risk of FOG and to monitor the efficacy of therapeutic strategies. Future longitudinal studies may support this assumption.

## Figures and Tables

**Table 1:** Biomechanical measurements. Abbreviations: AP, anteroposterior; C7, seventh cervical vertebra; CoM, center of mass; CoP, center of pressure; ML, mediolateral; PSIS, posterior superior iliac spine.

Description	Decomposition
**Anthropometric measurements (AMs)**	
Body Height [BH] (cm)	
Inter Anterior Superior Iliac Spine Distance [IAD] (cm)	
Limb Length [LL] (cm)	
Foot Length [FL] (cm)	
Body Mass [BM] (kg)	
Body Mass Index [BMI] (kg/cm^2^)	
**Base of support (BoS)**	
BoS Area (cm^2^): area of the polygon described by the markers placed on the heels, the lateral malleoli, the fifth metatarsal bones, and the hallux	
BoS Width (cm): distance between the ankle joint centers, estimated as the mid points between the lateral and medial malleoli	
Foot Alignment (cm): AP distance between the two markers placed on the heels	
βΔ: Difference between the left (βL) and right (βR) feet extra-rotation angles (angles between the axis passing through the lateral and medial malleoli and the horizontal axis of the reference system of the laboratory) (°)	
β: BoS opening angle, sum of βL and βR (°)	
**Postural angles**	
Angle between the line connecting the markers on the middle point between the PSIS and the C7 and the laboratory vertical axis (°)	
Angle between the line connecting the knee and hip centers of rotation and the laboratory vertical axis (°)	
Angle between the line connecting the knee and ankle centers of rotation and the laboratory vertical axis (°)	
**GI measurements—Imbalance (IMB)**	
IMB duration (s)	
IMB CoP displacement (mm)	AP, ML
IMB CoP average velocity (mm/s)	AP, ML
IMB CoP maximal velocity (mm/s)	AP, ML
CoM velocity at IMB end (m/s)	
CoM acceleration at IMB end (m/s^2^)	
CoP–CoM distance at IMB end (m)	
Orientation of CoP–CoM vector with respect to the progression line at IMB end (°)	
**GI measurements—Unloading (UNL)**	
CoP distance from the line passing through the markers on the heels at swing heel off (mm)	AP
UNL duration (s)	
UNL CoP displacement (mm)	AP, ML
UNL CoP average velocity (mm/s)	AP, ML
UNL CoP maximal velocity (mm/s)	AP, ML
CoM velocity at UNL end (m/s)	
CoM acceleration at UNL end (m/s^2^)	
CoP–CoM distance at UNL end (m)	
Slope of CoP–CoM vector at UNL end (°)	
**GI measurements—Stepping phase**	
CoP distance from the line passing through the markers on the heels at the swing foot toe-off (mm)	AP
CoM velocity at stance foot toe-off (m/s)	
CoM acceleration at stance foot toe-off (m/s^2^)	
CoP–CoM distance at stance toe-off (m)	
First step length (m)	
First step average velocity (m/s)	
First step maximal velocity (m/s)	

**Table 2:** Demographic, clinical, anthropometric, and base of support features. Data are shown as mean (standard deviation). No statistically significant difference was found across groups (Mann–Whitney U-test, *p*-value set at 0.05). Abbreviations: HC, healthy controls; LEDD, levodopa equivalent daily dose; PDF, Parkinson’s disease with freezing of gait; PDNF, Parkinson’s disease with no freezing of gait; UPDRS-III, Unified Parkinson’s Disease Rating scale, part III. Refer to Table 1 for a list of other abbreviations used.

		HC	PDNF	PDF
Demographic features	Gender (males/total)	14/23 (~61%)	10/20 (50%)	14/23 (~61%)
	Age (years)	61.17 (4.93)	63.32 (10.76)	63.83 (8.34)
Clinical data	Disease duration (years)	(-)	9.26 (3.89)	11.14 (3.47)
	Hoen and Yahr (I–V stage)	(-)	2.24 (0.42)	2.39 (0.50)
	UPDRS-III (0–108 score)	(-)	24.81 (9.43)	28.05 (9.96)
	LEDD (mg)	(-)	741.18 (221.26)	803.70 (358.33)
Anthropometric Measurements	BH (cm)	169.94 (10.53)	167.79 (11.05)	168.09 (11.44)
	LL (cm)	88.81 (5.37)	88.23 (7.81)	87.21 (5.84)
	FL (cm)	24.93 (1.66)	25.17 (1.58)	24.59 (1.65)
	BM (kg)	72.28 (11.11)	66.36 (13.01)	72.02 (14.83)
	BMI (kg/cm^2^)	24.59 (3.02)	23.00 (3.99)	24.89 (5.18)
	IAD (cm)	27.76 (2.35)	27.89 (2.53)	27.42 (3.40)
Base of Support	BoS area (cm^2^)	685.24 (91.56)	668.18 (75.19)	651.85 (114.90)
	BoS width (cm)	17.64 (4.10)	16.26 (2.78)	15.67 (2.59)
	Foot alignment (cm)	6.57 (3.36)	8.37 (4.54)	6.92 (3.76)
	Angle difference βΔ (°)	6.66 (3.29)	4.67 (2.58)	7.75 (4.98)
	BoS opening angle β (°)	40.67 (15.76)	37.25 (14.05)	43.56 (13.92)

**Table 4:** Postural angles were computed shortly before the gait initiation execution (during a 1 s window before the onset of the anticipatory postural adjustments). Data are shown as mean (standard deviation). Abbreviations: HC, healthy controls; PDF, Parkinson’s disease with freezing of gait; PDNF, Parkinson’s disease with no freezing of gait.

	HC	PDNF	PDF
Trunk (°)	4.08 ^#,+^ (2.43)	9.06 ^#^ (4.37)	12.58 ^+^ (5.65)
Thigh (°)	6.31 ^#,+^ (2.69)	0.54 ^#^ (3.70)	−0.48 ^+^ (4.00)
Shank (°)	9.22 (2.93)	10.67 (2.77)	10.95 (2.47)

Dunn’s test, significant *p*-value after Bonferroni correction: ^#^ HC vs. PDNF, ^+^ HC vs. PDF.

**Table 5:** Correlation between the shank angle and gait initiation measurements. Only significant partial correlations between postural angles and gait initiation measurements corrected for the influence of the group variable are shown (Spearman’s ρ, *p*-value < 0.05). No correlation was significant after Bonferroni correction for multiple comparisons.

		Spearman’s ρ	*p*-Value
Shank (°)	IMB average velocity (mm/s)	0.32	0.014
	IMB average velocity AP (mm/s)	0.31	0.016
	IMB maximal velocity AP (mm/s)	0.38	0.003

**Table 6:** Onset of segmental movements at gait initiation. Data are shown as mean (standard deviation). Time of movement onset of each segmental center of mass was expressed as the percentage with respect to total gait initiation duration (i.e., from the onset of the anticipatory postural adjustments to the heel contact of the swing foot) and compared across groups (Dunn’s test, no difference was significant after Bonferroni correction for multiple comparisons). Abbreviations: HC, healthy controls; PDF, Parkinson’s disease with freezing of gait; PDNF, Parkinson’s disease with no freezing of gait; ST: stance limb; SW: swing limb.

Segmental Center of Mass	HC	PDNF	PDF
Pelvis (%)	60.62 (9.27)	62.47 (13.74)	62.66 (21.69)
Thigh ST (%)	65.76 (10.75)	71.72 (20.13)	63.39 (19.97)
Shank ST (%)	71.17 (14.36)	68.57 (19.98)	65.73 (21.95)
Foot ST (%)	68.37 (10.46)	77.24 (22.53)	65.02 (22.87)
Thigh SW (%)	62.67 (9.23)	64.75 (15.47)	55.85 (18.80)
Shank SW (%)	74.21 (13.85)	75.35 (24.26)	67.26 (19.36)
Foot SW (%)	69.15 (15.41)	64.63 (16.55)	59.20 (19.86)
Chest (%)	73.48 (14.29)	74.47 (26.12)	69.22 (19.85)
Abdomen (%)	79.50 (13.87)	81.57 (25.30)	57.88 (20.58)
Arm ST (%)	55.49 (18.27)	65.80 (26.63)	54.17 (19.98)
Arm SW (%)	62.71 (9.34)	65.08 (13.49)	56.57 (17.72)
Forearm ST (%)	38.98 (12.65)	53.73 (24.00)	39.40 (12.69)
Forearm SW (%)	51.34 (11.80)	62.57 (13.59)	47.26 (13.34)
Hand ST (%)	44.37 (19.82)	49.64 (21.32)	41.99 (30.95)
Hand SW (%)	34.36 (13.06)	46.93 (19.96)	47.71 (16.69)
Head (%)	54.33 (11.81)	58.15 (14.06)	60.06 (18.41)

**Table 7:** Onset of rank-ordered segmental movements at gait initiation. Data are shown as mean (standard deviation). Time of movement onset of rank-ordered segmental centers of mass was expressed as the percentage with respect to total gait initiation duration (i.e., from the onset of the anticipatory postural adjustments to the heel contact of the swing foot) and compared across groups (Dunn’s test, no difference was significant after Bonferroni correction for multiple comparisons). Abbreviations: HC, healthy controls; PDF, Parkinson’s disease with freezing of gait; PDNF, Parkinson’s disease with no freezing of gait.

Rank-Ordered Segmental Center of Mass	HC	PDNF	PDF
1st segment (%)	27.88 (10.66)	35.42 (13.61)	26.51 (11.98)
2nd segment (%)	36.33 (10.40)	45.51 (15.10)	34.90 (13.48)
3rd segment (%)	42.62 (9.68)	50.70 (14.76)	41.43 (12.61)
4th segment (%)	46.70 (8.60)	54.47 (14.37)	44.29 (13.88)
5th segment (%)	50.58 (8.32)	57.41 (12.53)	46.68 (14.44)
6th segment (%)	54.40 (6.50)	59.49 (13.25)	49.54 (15.11)
7th segment (%)	57.49 (6.79)	61.70 (14.11)	51.87 (14.85)
8th segment (%)	59.71 (7.51)	64.81 (15.85)	54.13 (15.05)
9th segment (%)	61.31 (8.04)	66.18 (16.05)	55.93 (15.08)
10th segment (%)	62.94 (7.84)	67.59 (16.18)	58.10 (14.84)
11th segment (%)	65.75 (9.10)	68.86 (16.49)	60.79 (14.94)
12th segment (%)	67.89 (9.02)	70.65 (17.45)	62.66 (15.16)
13th segment (%)	70.64 (10.11)	72.32 (18.32)	65.83 (14.82)
14th segment (%)	73.91 (10.70)	74.85 (19.26)	68.93 (16.35)
15th segment (%)	78.42 (11.79)	78.81 (21.00)	74.20 (15.35)
16th segment (%)	84.95 (11.99)	83.19 (23.21)	82.42 (18.88)

## Data Availability

The data presented in this study are available upon request from the corresponding author. The data are not publicly available for privacy reasons.

## Supplementary Materials

The following supporting information can be downloaded at https://www.mdpi.com/article/10.3390/bioengineering9110639/s1, Figure S1: Rank order of segmental CoM onset times; Figure S2: Rank order of onset times of groups of segmental CoM.
