# Supplementary material for: Gait Initiation Impairment in Patients with Parkinson’s Disease and Freezing of Gait
Source: Bioengineering (Basel). 2022 Nov 2;9(11):639. doi: 10.3390/bioengineering9110639 (PMC9687939; doi:10.3390/bioengineering9110639)
Supplement: Supplementary file 1 [file bioengineering-09-00639-s001.zip › bioengineering-1966562-supplementary.pdf]

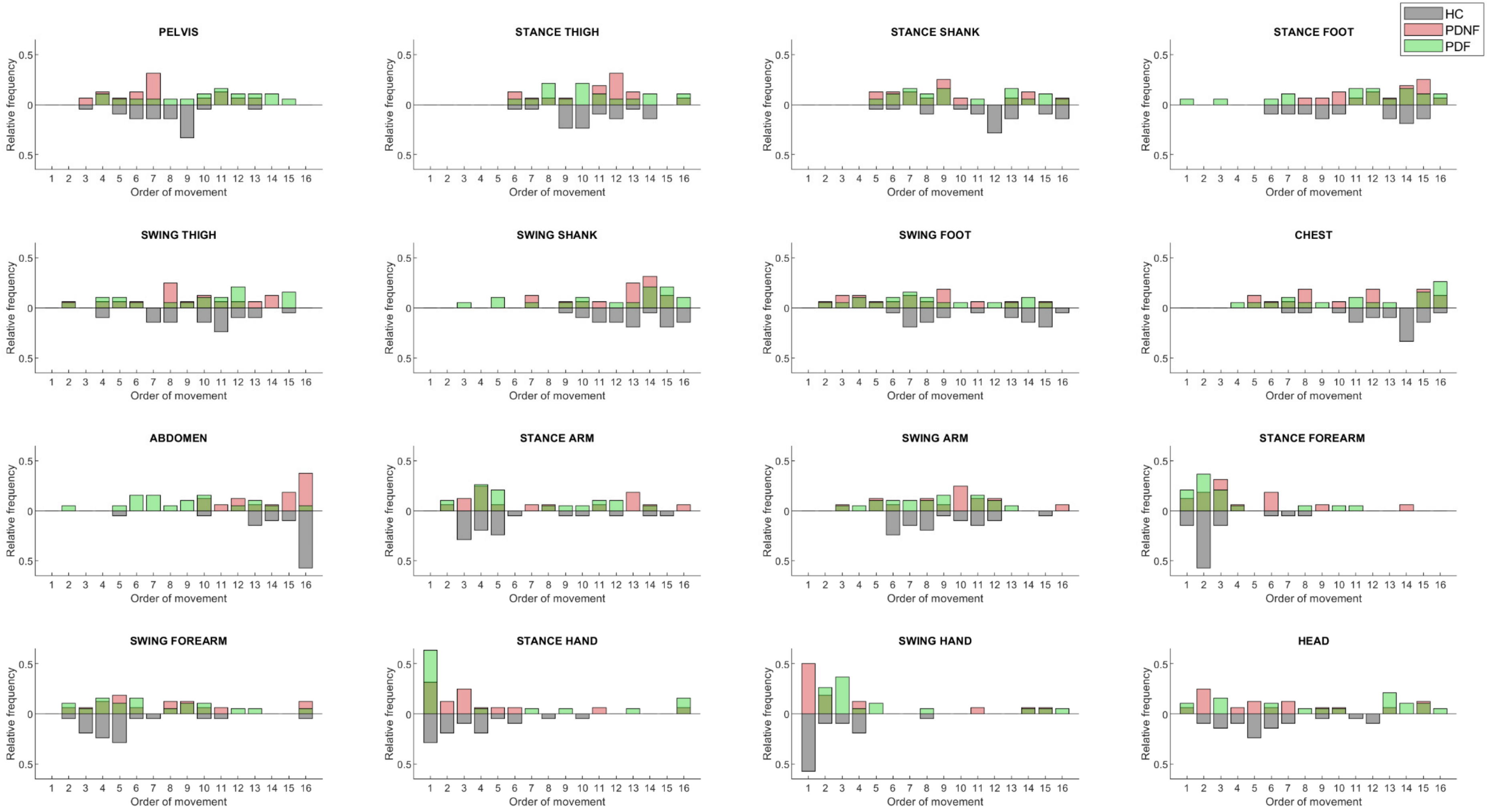

Figure S1. Rank order of Segmental CoM onset times. Temporal order of SCoM submovements within gait initiation for each group (grey: HC; red: PD; green: PDF). The bars represent the relative frequency with which each SCoM moves as 1-16th segment along GI execution. Abbreviations: HC, healthy controls; PDF, Parkinson's disease with freezing of gait; PDNF, Parkinson's disease with no freezing of gait; SCoM: segmental center of mass.

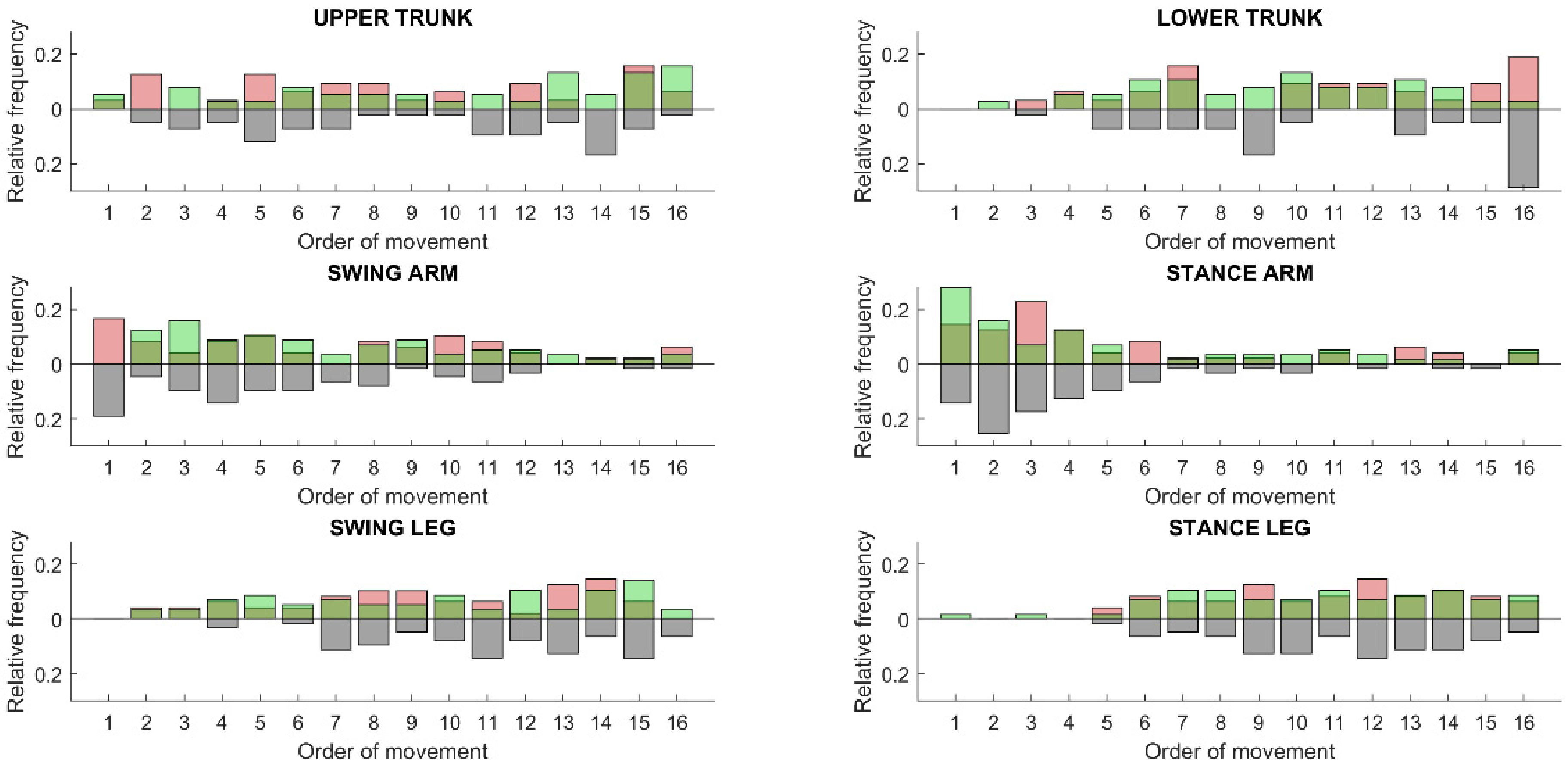

Figure S2. Rank order of onset times of groups of Segmental CoM. The rank ordered SCoM were classified into six groups (upper trunk: head and chest; lower trunk: abdomen and pelvis; swing arm: swing arm, forearm, and hand; stance arm: stance arm, forearm, and hand; swing leg: swing thigh, shank, and foot; stance leg: stance thigh, shank, and foot) and their temporal order of movement computed for each cohort (grey: HC; red: PD; green: PDF). The bars represent the relative frequency with which each SCoM moves as 1-16th segment along GI execution. Abbreviations: HC, healthy controls; PDF, Parkinson's disease with freezing of gait; PDNF, Parkinson's disease with no freezing of gait; SCoM: segmental center of mass.
